# Ecto- and endoparasite induce similar chemical and brain neurogenomic responses in the honey bee (*Apis mellifera*)

**DOI:** 10.1186/1472-6785-13-25

**Published:** 2013-07-17

**Authors:** Cynthia M McDonnell, Cédric Alaux, Hugues Parrinello, Jean-Pierre Desvignes, Didier Crauser, Emma Durbesson, Dominique Beslay, Yves Le Conte

**Affiliations:** 1 INRA, UR 406 Abeilles et Environnement, Site Agroparc, Domaine Saint-Paul, Avignon, Cedex 9, 84914, France; 2 Institut de Génomique Fonctionnelle, UMR 5203 CNRS, U661 INSERM, Universités Montpellier 1 & 2, Montpellier Cedex 05, 34094, France

**Keywords:** *Varroa destructor*, *Nosema ceranae*, Social immunity, Transcriptome, Cuticular hydrocarbons

## Abstract

**Background:**

Exclusion from a social group is an effective way to avoid parasite transmission. This type of social removal has also been proposed as a form of collective defense, or social immunity, in eusocial insect groups. If parasitic modification of host behavior is widespread in social insects, the underlying physiological and neuronal mechanisms remain to be investigated. We studied this phenomenon in honey bees parasitized by the mite *Varroa destructor* or microsporidia *Nosema ceranae*, which make bees leave the hive precociously. We characterized the chemical, behavioral and neurogenomic changes in parasitized bees, and compared the effects of both parasites.

**Results:**

Analysis of cuticular hydrocarbon (CHC) profiles by gas chromatography coupled with mass spectrophotometry (GC-MS) showed changes in honey bees parasitized by either *Nosema ceranae* or *Varroa destructor* after 5 days of infestation. Levels of 10-HDA, an antiseptic important for social immunity, did not change in response to parasitism. Behavioral analysis of *N. ceranae-* or *V. destructor-* parasitized bees revealed no significant differences in their behavioral acts or social interactions with nestmates. Digital gene expression (DGE) analysis of parasitized honey bee brains demonstrated that, despite the difference in developmental stage at which the bee is parasitized, *Nosema* and *Varroa*-infested bees shared more gene changes with each other than with honey bee brain expression gene sets for forager or nurse castes.

**Conclusions:**

Parasitism by *Nosema* or *Varroa* induces changes to both the CHC profiles on the surface of the bee and transcriptomic profiles in the brain, but within the social context of the hive, does not result in observable effects on her behavior or behavior towards her. While parasitized bees are reported to leave the hive as foragers, their brain transcription profiles suggest that their behavior is not driven by the same molecular pathways that induce foraging behavior.

## Background

Behavioral defenses are “strikingly effective” mechanisms for combating parasites but are often overlooked by studies addressing the effects of a parasite on the host immune system and related physiological processes [1]. For a behavior to be considered as an anti-parasite defense the parasite must impose a cost on the host and the behavior should limit or eliminate the parasite [2,3]. Parasite avoidance behavior is found across animal taxa, where it manifests as behaviors such as cleaning and avoiding feces or avoiding diseased conspecifics [4]. For example, normally gregarious spiny Caribbean lobsters shun lobsters that carry a lethal virus, even before the diseased lobster is visibly infected [5]. However, not all behaviors are anti-parasite, as parasites can also alter the behavior of their hosts in ways that are ultimately beneficial to the parasite or its offspring [6,7].

Living in groups can further complicate host-parasite interactions since group members can also play a role in the regulation of anti-parasite behavior. Collective defenses against parasites within a social group, called social immunity, are physiological, behavioral or organizational adaptations that prevent the transmission of the parasite [8,9]. A common social defense is the removal of the parasitized individuals from the social group. Indeed, in social insects, parasitized individuals can remove themselves from the group, which corresponds to an altruistic self-removal [10], or nestmates can modify their interaction with those individuals to prevent parasite transmission [6,9]. For example, in honey bees, very different types of stress, including exposure to the mite *Varroa*[11,12], the microsporidia *Nosema ceranae*[13,14], or immune challenge [15] have been shown to induce precocious foraging or forager-like physiological and behavioral characteristics. Leaving the colony to perform outside activities, like foraging, limits contact in the hive, especially with castes of great importance (e.g. queen, brood), and thus, the spread of parasites into the colony [9]. However, despite parasite modification of host behavior being widespread in animals, the underlying physiological and neuronal mechanisms are poorly understood, especially in social insects.

In order to better understand this phenomenon, we analyzed how two different parasites, an ectoparasite (*Varroa destructor*) and endoparasite (*Nosema ceranae*), affect the physiology and the brain neurogenomic state of honey bees. The *Varroa destructor* mite infects the larval cell immediately before capping where it feeds on the developing pupae and completes its reproductive cycle. It weakens the honey bee by feeding on its hemolymph and transmitting viruses, such as deformed wing virus (DWV), which are correlated with its effect on honey bee survivorship [16-18]. *Nosema* species are obligate, intracellular spore-forming fungal parasites that infect honey bee adults from emergence by spreading through the hive, most likely through the activities of cleaning and trophallaxis [19]. Once a worker has ingested *Nosema* spores, the spores develop in the intestine of the bee, where the germinated microsporidian infects the epithelial cell layer of the midgut and consumes the energy of the cell [20,21]. However, despite *Varroa* and *Nosema* infections varying greatly in their pathologies, they affect honey bee behavior and learning abilities in similar ways (see Table 1). For example, both *Varroa*-infested and *Nosema*-infected bees showed impaired orientation abilities at the hive entrance [22,23].

**Table 1:** **Comparative pathologies of *Varroa destructor *and *Nosema ceranae***

	***Varroa destructor***	***Nosema ceranae***
Type of parasite	ectoparasite	endoparasite
Mode of action	Sucks hemolymph/transmits viruses	Attacks epithelium of the gut
Mode of transmission	Enters brood cell before operculation; phoresis-carried between cells by adult bees (males and females)	Oral transmission between workers, matrices of the hive
Stage(s) attacked	Nymph/adult	Adult
Physiological effects	Reduced weight, metabolism, vitellogenin titers, and proportion of normal hemocytes; increased ecdysteroid titers [24,25]	Increase oxidative stress; degeneration of gut epithelium [26]; reduced vitellogenin level [27]; induces pheromone (ethyl oleate) production [28]
	Lifespan decrease [12]	Lifespan decrease
Behavioral effects	Impaired orientation [22]	Impaired orientation [29]
	Accelerated maturation [11,12]	Accelerated maturation [13,14]
	Faster habituation and lower response probability to odor stimulus but no change in sucrose response [30]	Increased sucrose response based on PER test [31]

Since there is a robust association between brain gene expression in the honey bee and its behavioral state [29,32,33], we measured the brain transcriptional changes induced by *V. destructor* and *N. ceranae* and determined whether they induce similar brain host responses. We also characterized the cuticular hydrocarbon (CHC) profiles of honey bees parasitized by *V. destructor* or *N. ceranae* and recorded nestmate interactions in observation hives in order to detect nestmate aggression toward parasitized bees. Indeed, challenging the immune system of bees with lipopolysaccharides or other non-living immune stimulants changed the CHC profiles of the bees, involved in social recognition, which lead to modified and aggressive conspecific contacts in a laboratory-based nestmate recognition assay [34,35]. Bees infected with the virus DWV, that showed changes in their CHC profiles, were also ejected from the hive at higher rates than healthy bees, notably from healthy hives [36]. Finally, we determined whether parasitism can affect the level of production of 10-HDA that contributes to social immunity of the colony. This compound, produced in the mandibular glands of bees, displays antiseptic properties in the royal jelly [37].

## Results

### Experiment 1: Chemical analysis of *Nosema ceranae*- or *Varroa destructor*-parasitized bees

#### Cuticular hydrocarbon profiles

Whether parasites induced changes in the cuticular hydrocarbon profiles (alkanes, alkenes, alkynes and methylalkanes) was tested in 11 to 12 bees per colony at days 5 and 10 post-emergence. The hydrocarbons were extracted from control and parasitized bees and analyzed and identified using gas chromatography (GC) followed by mass spectrometry (MS).

**Figure 1:**
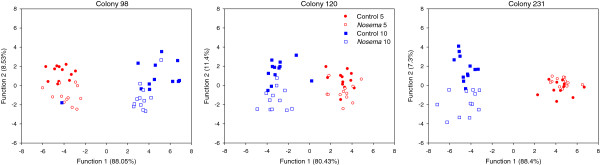
***Nosema*-infected bees developed different cuticular hydrocarbons profiles.** Discriminant analysis based on the cuticular hydrocarbons profiles of *Nosema*-infected and control bees at day 5 and 10. The analysis was repeated on bees from 3 different colonies (*N* = 11–12 bees/colony and treatment). Young (5 days) parasitized and control bees did not display different chemical profiles, but 5 days later both profiles were distinct (see Table 2).

We did not find new compounds in parasitized bees (*Nosema* and *Varroa*) as compared to control groups. Comparisons of the relative proportions of peaks corresponding to specific compounds did not reveal overwhelming differences between the *Nosema*-infected and control groups (Additional file 1: Table S1). However, the comparison of the overall chemical profiles, via discriminant analysis, of *Nosema*-infected bees and their control counterparts at days 5 and 10 showed highly significant differences for each colony (Colony 98: Wilks’ *λ* = 0.015, *F*_39,92_ = 7.39, *p* < 0.0001; Colony 120: Wilks’ *λ* = 0.011, *F*_39,.95_ = 8.73, *p* < 0.0001; Colony 231: Wilks’ *λ* = 0.037, *F*_27,102_ = 7.92, *p* < 0.0001; Figure 1). For each colony of origin, the squared Mahalanobis distance between *Nosema*-infected and control bees at 5 days old was not significant after Bonferoni correction (Table 2). Conversely, Mahalanobis chemical distances for older bees (10-day-old) were all statistically significant between infected and control bees (Table 2). The cuticular hyrocarbon profiles changed also with aging in both control and parasitized bees (Figure 1, Table 2). Cuticular profiles of worker bees were quite specific as it was possible to correctly assign between 72-100% of all workers from each age and infected groups (Additional file 2: Table S2).

**Table 2:** **Pair-wise squared Mahalanobis distances between the chemical profile of *Nosema*-infected and control bees at day 5 and 10**

**Colony #**	**Treatment**	***Nosema*5**	**Control10**	***Nosema*10**
Colony 98	Control5	7.2	68.62	47.92
		*P* = 0.023	***P*** **< 0.001**	***P*** **< 0.001**
	*Nosema*5		72.85	58.03
			***P*** **< 0.001**	***P*** **< 0.001**
	Control10			9.55
				***P*** **= 0.0055**
Colony 120	Control5	6.54	77.64	74
		*P* = 0.034	***P*** **< 0.001**	***P*** **< 0.001**
	*Nosema*5		77.93	77.45
			***P*** **< 0.001**	***P*** **< 0.001**
	Control10			9.59
				***P*** **= 0.0035**
Colony 231	Control5	4.28	31.22	34.92
		*P* = 0.044	***P*** **< 0.001**	***P*** **< 0.001**
	*Nosema*5		31.89	34.09
			***P*** **< 0.001**	***P*** **< 0.001**
	Control10			5.99
				***P*** **= 0.0057**

*P*-values that are significant at the 5% probability after Bonferroni's correction (*P* < 0.0083) are indicated in bold. In the treatment column, 5 and 10 indicate the age of the bees. The experiment was repeated on bees from 3 different colonies.

**Figure 2:**
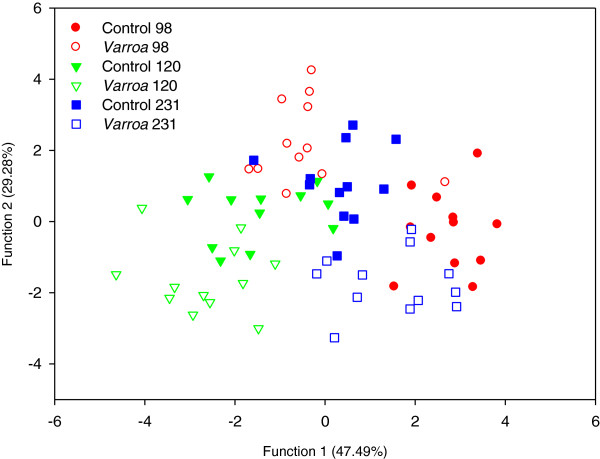
***Varroa*-infested bees developed different cuticular hydrocarbons profiles.** Discriminant analysis based on the cuticular hydrocarbons profiles of *Varroa*-infested and control bees. The analysis was repeated on bees from 3 different colonies (*N* = 12 bees/colony and treatment).

For *Varroa*-parasitized bees, the cuticular hydrocarbon profiles were compared with control bees on day 5 only (see Methods). The discriminant analysis also showed significant differences overall between parasitized and non-parasitized bees (Wilks’ *λ* = 0.027, *F*_75,253_ = 3.79, *p* < 0.0001, Figure 2). Mahalanobis chemical distances between *Varroa*-infested and control bees were highly significant and the colony of origin also had an important influence on the cuticular hydrocarbon profiles (Table 3). Between 75 and 100% of the individuals were correctly classified according to their chemical profiles (Additional file 2: Table S3).

**Table 3:** **Pair-wise squared Mahalanobis distances between the chemical profile of *Varroa*-parasitized and control bees**

**Treatment**	***Varroa*98**	**Control120**	***Varroa*120**	**Control231**	***Varroa*231**
Control98	17.46	18.02	28.31	9.61	6.2
	***P*** **< 0.001**	***P*** **< 0.001**	***P*** **< 0.001**	***P*** **= 0.0015**	*P* = 0.038
*Varroa*98		12.38	18.19	6.4	17.07
		***P*** **= 0.0012**	***P*** **< 0.001**	*P* = 0.032	***P*** **< 0.001**
Control120			9.09	8.34	17.7
			***P*** **= 0.0025**	*P* = 0.005	***P*** **< 0.001**
*Varroa*120				16.68	17.8
				***P*** **< 0.001**	***P*** **< 0.001**
Control231					9.72
					***P*** **= 0.0014**

*P*-values that are significant at the 5% probability after Bonferroni's correction (*P* < 0.0083) are indicated in bold. The numbers 98, 120 and 231 indicate the colony origin of the bees.

The relative proportion of each hydrocarbon affected by *Nosema* and *Varroa* is listed in (Additional file 1: Table S1). Some of them were significantly affected by the parasites and age but there was no consistent effect of age and parasite on the relative proportions of each compound.

#### 10-HDA levels

**Figure 3:**
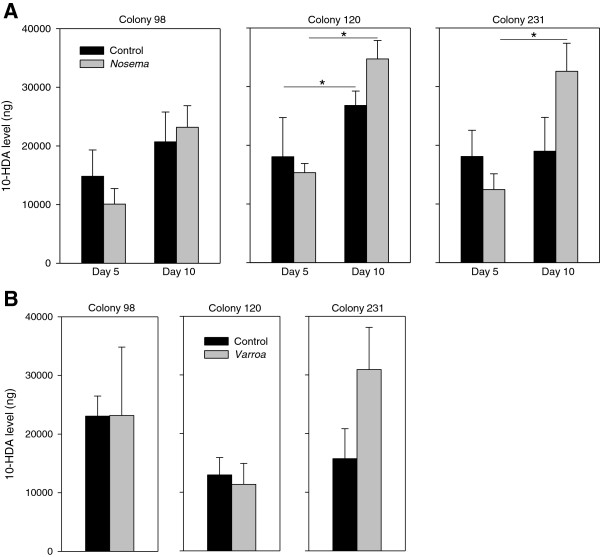
**Parasitism did not affect the levels of 10-HDA levels in (A) *Nosema*- or (B) *Varroa*-parasitized bees.** The 10-HDA levels did not differ between *Nosema*-infected and control bees at day 5 and 10 but increase with age in colony 120 and 231 (except for control) (Kruskall-Wallis tests: Colony 98: H = 4.53, *P* = 0.2; Colony 120: H = 19.32, *P* < 0.001; Colony 231: H = 8.57, *P* = 0.035; * denotes significant differences after Conover-Iman post-hoc tests, *P* < 0.05). *Varroa* did not modify the 10-HDA levels (Mann–Whitney tests: Colony 98: *P* = 0.38, Colony 120: *P* = 0.68; Colony 231: *P* = 0.058).

We compared 10-HDA levels in the heads of *Nosema*- and *Varroa*-parasitized bees to control bees from the same colony (Figure 3A and B). For the comparison of *Nosema*-parasitized and control bees, there was no significant difference in 10-HDA levels, but for two colonies 10-HDA showed a significant increase with age (Figure 3A). Levels of 10-HDA did not differ between *Varroa*-parasitized and control bees (Figure 3B).

### Experiment 2: Behavioral analysis of *Nosema ceranae*- or *Varroa destructor*-parasitized bees

**Figure 4:**
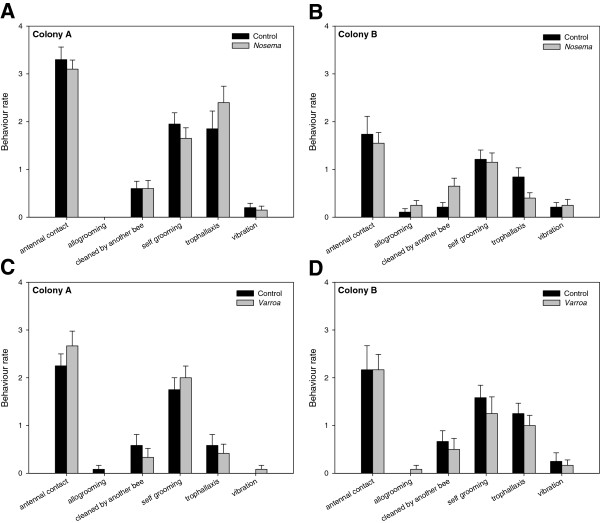
**Parasitism did not induce different behavioral treatment by colony nestmates.** The rate of behavioral acts or interactions did not differ between *Nosema*-infected and control bees (**A** and **B**), and *Varroa*-infested and control bees (**C** and **D**) (Mann–Whitney tests, *P* > 0.05 for each behavior). The experiment was performed on two colonies (19–20 bees/state in the *Nosema* experiment and 12 bees/state in the *Varroa* experiment).

In two four-frame observation hives, we quantified a suite of social behaviors (antennal contact, allogrooming, self-grooming, cleaned by another bee, trophallaxis and vibration) undertaken or received by *Nosema*-parasitized (*N* = 40) and control bees (*N* = 39). No significant differences were observed between parasitized and healthy bees in the rate of behavioral acts or social interactions (Figure 4A and B). Similarly, *Varroa*-parasitized bees did not display different behavior and were not treated differently by nestmates as compared to control individuals (*N* = 24 for each group) (Figure 4C and D). In addition, we did not see any agonistic behavior toward parasitized bees during the observation periods (600 min for *Nosema*-parasitized and 585 min for control bees; 360 min for both *Varroa*-parasitized and control bees).

### Experiment 3: Brain transcriptomics of *Nosema ceranae*- or *Varroa destructor*- parasitized bees

The brain transcriptome modifications induced by parasitism were determined in bees originating from two different colonies using digital gene expression (DGE) analysis. Two DGE-tag libraries were generated for each experimental group, control, *Nosema*-infected and *Varroa*-infested. For each library more than 100,000,000 tags were produced that were then narrowed down to around 9,300 unique gene hits for the honey bee (Additional file 3: Table S4). These distinct tags and their genomic count are available from NCBI Gene Expression Omnibus (GEO) database with the accession number: GSE41109.

The number of genes whose expression was affected in the bee brain by *Nosema*- and *Varroa*-parasitism was markedly different. In *Varroa*-infested bees 455 genes changed overall (225 up- and 230 downregulated) while in *Nosema*-infected bees only 57 genes responded differentially (12 up- and 45 downregulated) at an adjusted *P*-value < 0.05 (Additional file 4: Table S5).

Gene Ontology analysis revealed that gene expression changes in *Varroa*-infested brains (represented by 265 fly orthologs) were most significantly overrepresented in the metallopeptidase functional group with changes in both directions (Table 4). *Varroa*-infested bees showed decreased expression of glutamate and GABA receptor-related genes (GB15851, GB14954, GB13604, GB15167, GB18621), and the dopamine receptor, *Amdop1*, and overexpression of ascorbate/aldarate metabolism genes (GB17015, GB16747, GB14956, GB15000), which include dopamine and serotonin metabolism [38-40]. Conversely, both glutamate decarboyxlase 1, which synthesizes GABA neurotransmitter, and the GABA neurotransmitter transporter 1B were overexpressed in *Varroa*-parasitized brains (Table 4). *Nosema*-infected brains, for which only 24 genes had Flybase orthologs, showed no significant patterns in the classification of functional groups. Several genes that are involved in immune and antioxidant activity, defensin-1, peroxidase, esterase A2, glucose oxidase, flavin-containing monooxygenase, were upregulated.

**Table 4:** **Functional analysis of genes regulated by *Varroa *parasitism**

**Biological process/ Molecular Function**	***P*-value**	**# upregulated genes**	**# downregulated genes**
Metallopeptidase activity	0.00193	5	4
Ascorbate and aldarate metabolism	0.0106	4	0
GPCR, family 3, C-terminal	0.0126	0	3
Metalloendopeptidase activity	0.0127	2	4
Nucleophile	0.0150	2	3
Carbohydrate binding	0.0170	2	5
Integral/intrinsic to plasma membrane	0.0243	4	6
Glutamate receptor activity	0.0320	0	4
Pattern/ Polysaccharide binding	0.0331	1	4
Starch and sucrose metabolism	0.0338	3	1
Peptidase activity, acting on L-amino acid peptides	0.0379	10	5
Hydrolase	0.0431	20	16
Developmental growth	0.0471	3	3
Metalloprotease	0.0482	2	2

Gene ontology biological process and molecular function categories that were overrepresented in the brain transcriptomic profile are shown (*P* < 0.05).

**Figure 5:**
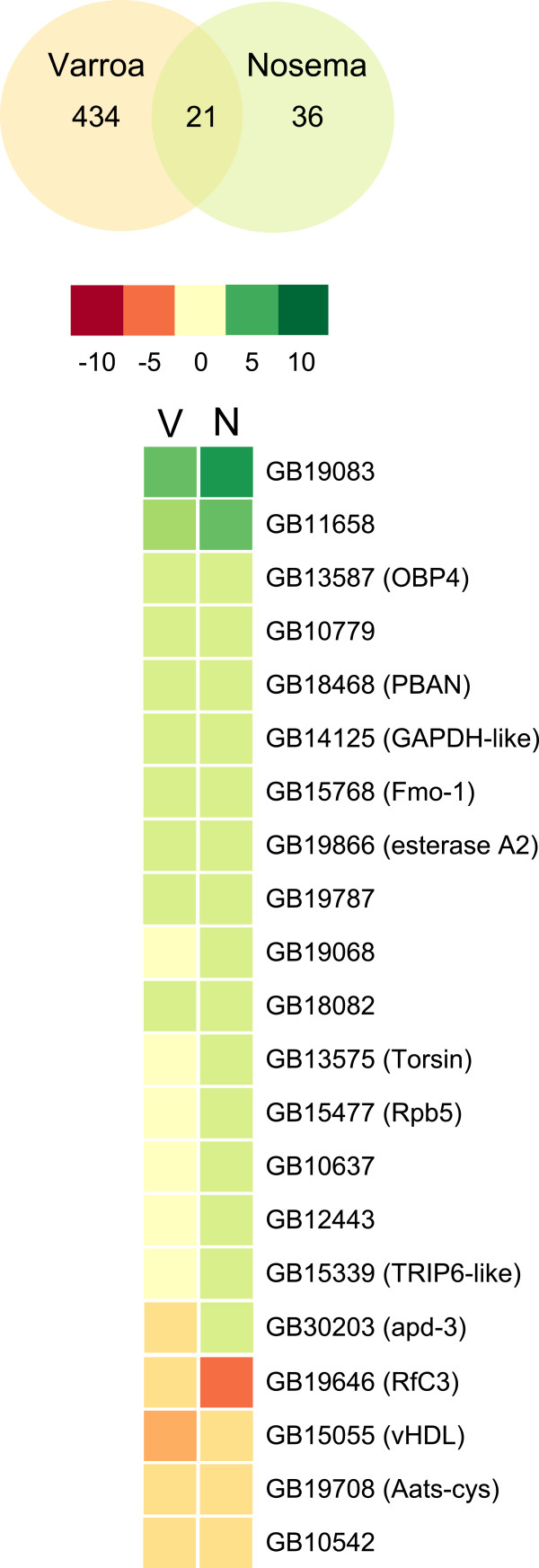
**Expression levels show similar directionality for 20 brain genes commonly affected by *Nosema *and *Varroa *parasites.** For the total number of genes changed by parasitism by *Varroa* (*N* = 455 genes) or *Nosema* (*N* = 57 genes), a statistically significant number of genes occur in both lists (*N* = 21) with 20 genes expressed in the same direction (Exact hypergeometric probability test: *P* < 0.0001). Color scale for the heatmap (red to green) indicates log2 transcription ratios where red color indicates underexpression of the gene in the parasitized bee and green color indicates overexpression. For each gene, the accession number and annotation are indicated.

Despite the difference in overall number of genes affected by both parasites as compared to controls and the 245 genes that changed between *Varroa* and *Nosema* parasitism (Additional file 4: Table S5), *Nosema*- and *Varroa*- parasitized bees shared more gene changes with each other (21 genes, Figure 5) than expected by chance (5.6 times more genes). In addition, *Nosema* parasitism caused a brain gene expression profile that was similar to the profile of bees parasitized by *Varroa*; except for apidermin 3 (GB30203), genes that were up- and downregulated by *Nosema* were also up- and downregulated by *Varroa,* giving a significant pattern (*χ2* = 11.049, *P* < 0.001, Figure 5). We also tested gene expression overlap with brain gene expression data from nurse/forager, i.e. genes known to be upregulated in nurse brains as compared to forager brains and the other way round [41]. We found respectively 8 and 34 genes affected by *Nosema* and *Varroa* parasites to overlap with the nurse/forager sets (Additional file 4: Table S5) but neither of these gene sets was significantly similar to nurse and forager bees (*χ2* = 0.08, *P* = 0.78 and *χ2* = 0.58, *P* = 0.45 for *Nosema* and *Varroa*, respectively).

**Figure 6:**
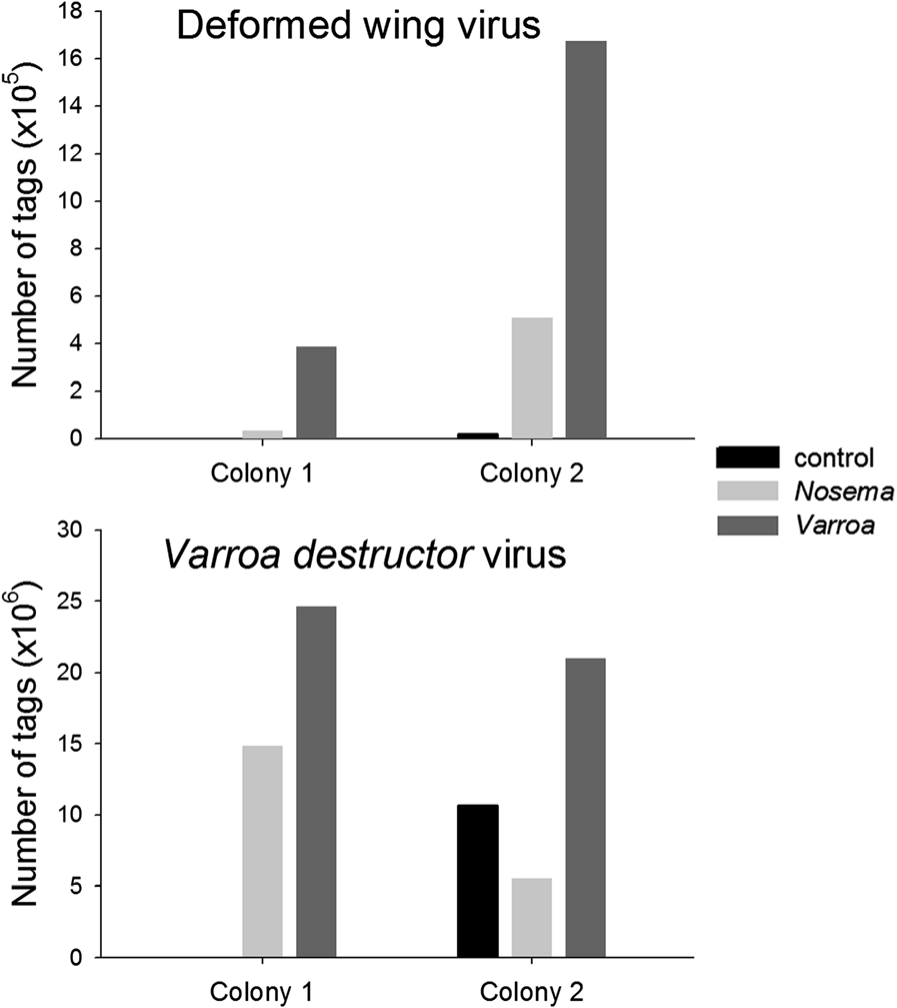
**Deformed wing virus titers increased in the honey bee brain with parasitism.** Viral titers are expressed as the total number of tags in a sample for each colony individually (Colony 1 and Colony 2). Deformed wing virus (DWV) levels were significantly higher in *Varroa*-infested bees compared to *Nosema*- and control bees (Generalized Linear Model with Cox-Reid method for estimating dispersion; *Varroa* vs. control: *P* < 0.05, *Varroa* vs. *Nosema:P* < 0.01), while increased DWV levels in *Nosema*-infested bees were marginally significant (*P* < 0.10). *Varroa destructor* virus (VDV) titers did not differ between parasitized and control bees (For *P*-values see Additional file 5: Table S6.)

Using the DGE-tag libraries, we determined whether parasites affected the viral landscape in the bee brain. We looked for presence and abundance of 9 viruses: Chronic bee paralysis virus RNA 1, Chronic bee paralysis virus RNA 2, Sacbrood virus, Deformed wing virus, Black queen cell virus, Acute bee paralysis virus, Kashmir bee virus, Varroa destructor virus 1 and Israel acute paralysis virus. Only two viruses were identified in the bee brains, Deformed wing virus (DWV) and *Varroa destructor* virus (Figure 6). *Varroa*-infested bees had the highest levels of DWV compared to control bees and higher levels of Deformed wing virus than *Nosema*-infected bees (Figure 6, Additional file 5). *Nosema*-infected bees also had higher levels of DWV than control bees, at the limit of statistical significance. *Varroa destructor* virus levels were not statistically different across control, *Varroa-* and *Nosema-* parasitized bees.

## Discussion

In this study, we demonstrated that two parasites, *Varroa destructor* and *Nosema ceranae*, with distinct, differing pathologies both modified the physiology and transcriptomic profiles in the brain of their honey bee host. Parasitized honey bees exhibited changes in their CHC profiles but showed no differences in behaviors between parasitized and healthy bees. In addition we observed no significant aggressive behavior towards parasitized bees, nor any change in social interactions.

A previous study found that bees parasitized by *Varroa* exhibit a modified CHC at their emergence [42]. Our data shows that this change is lasting. However, our behavioral results differ from recent studies that observed increased general social interactions or aggressive behaviors towards immune-challenged bees, but differences in the experimental design of our study may account for those differences. In a series of studies, increased social and aggressive behaviors towards immune-suppressed workers were observed a few hours after treatment in assays that were conducted in the laboratory [34,35]. Our study employed natural conditions of a four-frame observation hive using bees that had been parasitized in the days prior to the experiment, in order to understand if nestmates respond to parasitized bees within the context of general activity of the hive. Moreover we chose to focus on bees that were *Varroa*-parasitized but asymptomatic for DWV. In contrast, a second study found that DWV-infected bees that exhibited deformed wing symptoms were detected and removed from the hive by nestmates [36]. Thus our results do not contradict previous studies but reflect the subtle nature of parasitism by *Varroa* or *Nosema* that, when resulting in precocious departure from the hive, is more likely due to altruistic self-removal, acting as a mechanism of social immunity. Indeed, it may be less costly for the colony that sick or parasitized bees leave the colony of their own accord, rather than recruiting nestmates to exclude those bees via aggressive behaviors. In that case, bees might distinguish sick bees based on different CHC profiles but not discriminate them, except in the case of extremely sick bees that cannot leave on their own, such as bees exhibiting deformed wing symptoms. We also found a change of the chemical profile with age which is consistent with previous studies (nurse vs forager, see [43]). In honey bee hives, older bees segregate themselves from young bees by olfactory discrimination of cuticular hydrocarbons as they correspond to different age groups [44]. Indeed, older bees both emit and respond to a more complex bouquet of cuticular hydrocarbons than younger bees [43,44]. Since *Nosema* and *Varroa*-parasitized bees age faster, it is possible that they exhibited a CHC profile of old bees. In addition, the CHC profile is shaped by the genotype, nutrition, environment and physiological state [45,46]. Therefore, it is possible that their nestmates did not respond to the parasitized bees because their chemical profiles could not be distinguished from the chemical profile of others bees of different ages, physiological status and genotypes. These results highlight the importance of testing for biological effects within the hive when trying to draw conclusions about honey bee behavior.

If parasitism by *Varroa* or *Nosema* induces precocious foraging, one would expect the parasitized bees to show physiological changes similar to the transition to a forager bee. Levels of 10-HDA increased with the age of the bee confirming the study of Plettner *et al.*[47], but did not change in response to *Nosema* or *Varroa* parasitism. Thus the production of antiseptic compounds, like 10-HDA, in the food is age- or task-dependent but not regulated by the presence of parasites. However, further investigation of different type of pathogens or parasites, would give more insight as to whether antiseptic production can vary according to the infection level of the hive.

Our results also demonstrate that parasites alter the brain of the honey bee host, whether they were parasitized at the pupal (*Varroa*) or the adult stage (*Nosema*). In addition, twenty genes, nearly one half of those detected in *Nosema*-infested bee brains, show a shared expression pattern between *Varroa* and *Nosema*-infested bees. Their functions are diverse but several genes stand out as interesting for their possible roles in oxidative stress, neural function and foraging behavior. *Flavin-containing monooxygenase FMO GS-OX-like 3-like* (*FMO3*) and *torsin-like protein* (*torp4a*) are overexpressed and *replication factor C subunit 5-like* (*RfC5*) is downregulated in *Varroa*-infested and *Nosema*-infected bees. FMO3 is part of the FMO family known to react to xenobiotic stress in other organisms [48]. In *Drosophila*, the *Torp4a* ortholog (*dtorsin*) is involved in dopamine metabolism and locomotion [49] and the *RfC5* ortholog (*RfC3*) plays a role in neurogenesis [50], indicating that both parasites can modify brain function. The honey bee gene, *Pheromone biosynthesis-activating neuropeptide* (*PBAN*) is also over-expressed in both *Varroa*-infested and *Nosema*-infected bees compared to controls. In honey bees, *PBAN* neuropeptide levels are significantly higher in nectar foragers than pollen foragers [51]. In Lepidoptera, *PBAN* is linked to regulation of sex pheromone production [52], where pheromone production is JH-dependent, and JH primes the pheromone gland in adult females to respond to *PBAN*[53]. JH is also responsible for “priming” the foraging behavior in honey bees, though no link has been established between *PBAN* and JH in honey bees.

The presence of viruses may also account for similarities in gene expression between *Nosema* and *Varroa*-parasitized bees. We found increased levels of DWV in the brains of both types of parasitized bees and therefore cannot exclude that the observed changes are actually caused by an increase in DWV titer. DWV is a positive-strand RNA picorna-like virus that has been detected and can actively replicate in the heads [54] and brains of honey bees, specifically the mushroom bodies, visual and antennal lobe neuropils [55]. The virus is closely associated with *Varroa* infestation [56] and thus it was not unusual that it occurred in higher levels in *Varroa*-infested brains. On the other hand, we did not expect to find an increase in DWV levels in *N. ceranae*-infected bees, as a negative correlation between these two pathogens in the midgut of the bee was recently reported [57], which suggests that *N. ceranae* and DWV may compete for resources in the degenerated midgut, but not in the brain, where *Nosema* is not found.

Despite the statistically significant number of shared genes that change, *Varroa* and *Nosema*-infested brains demonstrate different patterns of expression that may reflect the different pathologies of the two parasites. Bees that were parasitized by *Varroa* as developing pupae exhibit more gene changes compared to controls than bees that were inoculated with *Nosema ceranae* as one-day old adults. This apparent disparity in gene expression changes may be due to long-lasting brain developmental changes induced during pupal development that persist in adult bees. The genes affected by *N. ceranae* infection could not be sorted by functional group analysis but several immune-related and antioxidant genes, including *defensin-1*, *peroxidase*, *esterase A2*, *glucose oxidase*, were upregulated indicating that the blood–brain barrier in honey bees, although not well studied, may be compromised by a parasitic attack. Genes involved in the oxidative response to stress were also upregulated in the guts of *N. ceranae*-infected honey bees [58], suggesting a systemic response throughout the honey bee in response to the microsporidian.

The impact of *Varroa* on the brain transcriptome suggests a decrease in learning and memory that may result from parasitism during development. This brain response would explain the actual learning impairment and losses of foragers induced by *Varroa*[23,59]. Based on functional group analysis, *Varroa*-infested bees show decreased expression of glutamate and GABA receptor-related genes, the dopamine receptor, *Amdop1*, and overexpression of ascorbate/aldarate metabolism genes. Inhibition or suppression of glutamate receptors disrupts memory formation in honey bees [26,30,60]. GABA receptors are present throughout the mushroom bodies [61], a region important for learning and memory, especially in foragers [62]. GABAnergic interneurons also form part of the olfactory conditioned learning pathway [63]. Yet the simultaneous increase in the expression of glutamate decarboxylase and GABA neurotransmitter transporter with a decrease in GABA receptor targets signals either compensatory mechanisms at work or a disruption in GABAnergic network. Analysis of the neuroanatomical changes in the *Varroa*-parasitized brain could resolve whether decreased expression of GABA and glutamate receptors leads to a reduction in their numbers. The dopamine receptor *Amdop1* is higher in newly born cells in the mushroom body than older cells [64] and in *Drosophila*, it is required for aversive and appetitive learning [65]. Finally, the cAMP pathway and its targets in the mushroom bodies are important mechanisms for learning in bees [66]. Several genes linked to the cAMP and calcium signaling cascades are downregulated in *Varroa* brains: *Adenylate cyclase type 10-like*, *Ryanodine receptor*[67] and *voltage-dependent calcium channel subunit* (GB10696).

Compared to the transcriptomic changes in the honey bee brain that accompany the switch from nurse to forager, we found relatively few genes that changed in response to *Nosema ceranae* or *Varroa destructor* infestation. Brain expression profiles of *Varroa* and *Nosema*- parasitized bees bear a greater resemblance to each other than to the reported profiles of typical foragers or nurses. Thus, their early departures from the hive may not be induced by mechanisms of normal behavioral development, but perhaps by an alternative mechanism that results in altruistic self-removal. Indeed, certain genes that are typically upregulated in foraging bee brains (*Inos*, *Kr-h1*) are downregulated in *Varroa*-infested honey bee brains [29,41,68,69]. Foraging activity is an especially demanding activity for learning and memory in the honey bee [63], but parasitized bees seems to have deficiencies at this level (see above). Therefore, altogether these results suggest that *Varroa* and *Nosema*-parasitized bees seem to not be true foragers, much like CO_2_-treated bees that left the hive, but also disappeared, at higher rates than control bees [10]. However, this will require confirmation in a more natural context (colony level).

Neither of the parasites, *Varroa destructor* nor *Nosema ceranae*, attacks the honey bee brain directly, yet we observed transcriptional changes in the brains of honey bees in response to parasitism. Thus, these changes, that are most likely triggered by a reaction in another tissue (e.g. midgut, fat bodies, hemolymph), highlight a link between the immune system, the brain, and perhaps, behavior in the honey bee. While parasitized bees are reported to behave like foragers, by leaving the hive, their brain transcription profiles suggest that their behavior is not driven by the same molecular pathways that induce foraging behavior. Whether the transcriptional changes observed are due to host immune response, parasite protein release or viruses that propagate in the brain is not known. LPS-challenged bees also behave more like foragers than same-aged bees [15], even without parasitic or viral challenges, but proteomic analysis of parasitized insects, grasshopper (by a nematode) and tsetse fly (by *Trypansoma brunei*) detected changes in the host brain [70,71] and proteins released by the parasite that may affect host behavior [70].

## Conclusion

Stress response in the honey bee to parasitism by *Varroa destructor* or *Nosema ceranae* shows similarities in their features; both parasites induce changes in CHC profiles and similar transcriptional profiles in the brain. That these parasitized bees are not attacked by their nestmates suggests that they leave the hive voluntarily, perhaps propelled by gene expression changes in the brain, showing altruistic behavior as predicted by Rueppel *et al.*[10]. This social removal may be a general and conserved response to parasitism, given that it was observed with extremely different types of parasites: a mite (ectoparasite) [11,12] and a single cell microsporidian (endoparasite) [13,14]. As to what these bees do once they have left the hive still needs to be examined. Are they normal foragers but with shorter life spans, less efficient foragers due to learning and memory deficiencies or do they leave the hive and wander aimlessly in the landscape? Emerging tracking technology will allow us to answer these questions and determine the role of the parasitized honey bee within the colony. In addition, such studies that incorporate behavioral, genomic and physiological components will help us to better understand current worldwide declines of honey bee populations that are often characterized by an unusual loss of adult bees from the colony [72].

## Methods

### Bees and parasites

This experiment was performed using hives of a hybrid of *Apis mellifera mellifera* and *A.m. ligustica* located at the Institut National de la Recherche Agronomique in Avignon, France. *Nosema*-treated bees were individually fed 2 uL of 50% sugar solution with a mixture of freshly extracted spores of *Nosema ceranae* at a concentration of 50,000 spores/uL. The presence of *N. ceranae* was confirmed by PCR analysis [73]. Guts were dissected at the end of the experiments and no spores were found in the control bees (data not shown). *Varroa*-parasitized bees were obtained following a similar procedure described in [74]. Colonies that were not treated with miticide were used and the queen was caged to stop egg-laying so that *Varroa* mites had no cells to parasitize. Meanwhile, the queen from a different colony was transferred into a queen-excluder for 7 days with an empty frame. Then the frame containing new brood (young larvae) was transferred into the colony that had a caged queen and sealed brood. The new brood on this frame was then uniformly parasitized by *Varroa* mites. Three weeks after the queen laid eggs in the frame enclosed in the queen-excluder, the frame was removed from the colony and newly emerging adults were picked from capped cells in order to verify whether the cell was infested with *Varroa*. *Varroa*-parasitized bees with deformed wings were discarded due to their extremely short lifespan.

### Experiment 1: Chemical analysis of *Nosema ceranae*- or *Varroa destructor*-parasitized bees

Experiments on *Nosema* and *Varroa* were performed separately but following the same procedure. In each experiment bees from three colonies were used. One day after their emergence, parasitized and non-parasitized (control) bees (*N* = 40–60 bees/state/colony) were color painted (Lackstift, Motip, Netherlands) on the thorax according to their state and colony origin and then all transferred into a host colony that was *Varroa*-treated and *Nosema*-free. At this age bees are easily accepted by the colony since they are lacking the recognition cues, including cuticular hydrocarbons [75]. The hydrocarbon profiles, which are genetically and environmentally acquired, change progressively with age [75,76]. After 5 and 10 days in the *Nosema* experiment, and after 5 days in the *Varroa* experiment, bees were collected and stored at −80°C for later chemical analysis. *Varroa*-parasitized bees were not collected at day 10, because they were more difficult to find in the hive at that age and in sufficient number for later analysis (shorter lifespan or had left the hive).

#### Cuticular hydrocarbon profiles

Hydrocarbons were extracted by individually immersing bees for 5 minutes at room temperature in 1,900 μL of isohexane and 100 μL of eicosane (C20) at 25 ng/μL as an internal standard. Each sample was concentrated under a stream of nitrogen to a volume of 10 μL, of which 1 μL was injected into a fast gas chromatograph (GC) (Shimadzu 2014, Japan) equipped with a split-splitless inlet, a flame ionization detector, and a capillary column Equity 5 (15 m x 0.10 mm, 0.10 μm film thickness). Samples were injected in split mode and hydrogen was used as a carrier gas with a column flow of 0.55 ml/min. The oven temperature was held at 70°C for 30 sec., increased from 70°C to 150°C at 40°C/min., from 150°C to 320°C at 10°C/min., and held at 320°C for 10 min. Peaks from the paint were identified and automatically removed by comparing the profile of non-marked bees to paint-marked bees and paint diluted in isohexane.

The structure of cuticular compounds present in the profiles was determined by performing gas chromatography coupled with mass spectrometry (GC-MS). Two μL of sample was injected into a GC-MS Thermo Scientific Trace GC Ultra ISQ equipped with a split-splitless inlet, an ISQ electron impact ion source, and a Thermo TR-5 column (20 m x 0.10 mm, 0.10 μm film thickness). The column flow was 0.4 ml/min. and the oven temperature was held at 50°C for 43 sec., increased from 50°C to 150°C at 20°C/min., from 150°C to 300°C at 10°C/min., and held at 300°C for 10 min.

For statistical analysis of the chemical profiles, only peaks that were reproducibly quantifiable in all samples were used. Each peak area was standardized according to Reyment [77]. To determine whether parasitized and control bees could be distinguished on the basis of their cuticular profiles and assess the profile similarity, a stepwise discriminant analysis was performed with Statistica 8.0. (StatSoft® Inc.). In addition, Mahalanobis distances between all pairwise groups were calculated as estimates for the chemical distances between each group. *P*-values were adjusted for multiple comparisons using Bonferroni’s correction. The effect of parasitism on the relative proportion of each compound was determined by using Mann–Whitney U tests.

#### 10-HDA levels

Chemical compounds were extracted by crushing individual heads in 200 μL of methanol and 100 μl of decanoic acid (250 ng/μl; internal standard) for 2 min. 30 sec. at 50 Hz with a Mini-Mill Pulverisette 23 (Fritsch, France). The solution was centrifuged at 4,000 g for 40 min. Twenty μL of the supernatant were collected, concentrated under nitrogen stream and then derivatized with 5 μL of bistrimethylsilyltrifluoroacetamide. The solution was agitated and left at room temperature for 40 min. The derivatized sample was then diluted in 10 μL of isohexane and 1 μL of this solution was injected into the GC (Shimadzu 2014, Japan). The samples were injected in split mode. Hydrogen was used as carrier gas. Oven temperature was set at 100°C, then increased to 200°C at 40°C/min. and to 250°C at 10°C/min. and held at 250°C for 2 min. Identification and quantification of 10-HDA were based on retention times of synthetic compounds (Cayman Chemical, France). The confirmation of 10-HDA compound was done by mass spectrometer (Thermo Scientific Trace GC Ultra ISQ). *Nosema* and *Varroa* effects on 10-HDA synthesis were determined by using Kruskall-Wallis and Mann–Whitney U tests, respectively.

### Experiment 2: Behavioral analysis of *Nosema ceranae*- or *Varroa destructor*-parasitized bees

To determine whether parasitized bees are treated differently than healthy, control bees, we recorded social interactions between focal bees and nestmates in two four-frame observation hives. *Nosema-* (70 bees/state/observation hive) and *Varroa*-parasitized bees (30 bees/state/observation hive) were obtained as previously described, tag-numbered (Opalith Plättchen, Friedrich Wienold, Germany) and introduced into the observation hives. The two experiments were carried out separately. We performed focal sampling behavioral observation on randomly-picked bees for 15 min (19–20 bees/state/observation hive for the *Nosema* experiment and 12 bees/state/observation hive for the *Varroa* experiment). The regular behaviors recorded during the observation period were: antennal contacts, allo-grooming, cleaned by another bee, self-grooming, trophallaxis and being vibrated (vibration signal, see [78]). The agonistic behaviors were: mandibular openings, bites, and stinging. We then determined whether the rate of each behavior performed during the observation period differed between parasitized and non- parasitized bees using Mann–Whitney U tests.

### Experiment 3: Brain transcriptomics of *Nosema ceranae*- or *Varroa destructor* -parasitized bees

Three treatment groups were used: control bees that had no presence of *Varroa* in their brood cells, *Varroa*-infested bees that had *Varroa* in their cells, and *Nosema*-infected bees that received the *Nosema* sugar solution. Each treatment group was obtained as previously described (see *Bees and parasites*) and then each group composed of one day old bees (*N* = 20 bees/treatment group) were housed in different plastic cages (10.5 × 7.5 × 11.5 cm). Keeping the bees in cages allowed us to remove the potential effect of hive environment and capture only the effect of the parasite on brain gene expression. The experiment was repeated on 2 different colonies.

#### Brain dissection

After 10 days in cages, bees were sacrificed by flash freezing in liquid nitrogen. Heads of the bees were separated from the body and the cuticle scratched with surgical tweezers before storing in RNA*later*®-ICE solution (Life Technologies) at −20°C for 16–18 hours, according to manufacturer’s instructions. Brains were dissected on ice under a dissection microscope to remove all traces of the optic lobe and then stored at −80°C.

#### RNA isolation

For each cage, 3 pools of 3 bee brains were homogenized in 500 μL of TRIzol Reagent (Life Technologies), phase-separated with chloroform/Trizol and the aqueous phase removed for precipitation with 70% ethanol. The resulting aqueous-ethanol solution was loaded onto an RNeasy mini spin column of the Qiagen RNeasy Mini Kit (Qiagen). RNA isolation was performed according to the manufacturer’s instructions, starting with washing with Buffer RW1. Genomic DNA was removed from samples using an RNA-free DNase set (Qiagen). RNA was quantified by spectrophotometry using the Nanodrop 1000 (Thermo Scientific). Then, RNA isolated from the 3 pools was equally combined.

#### Digital gene expression

For each treatment, two brain pools (one per colony) were analyzed. Sample preparation was performed using the DGE DpnII Sample preparation kit (Illumina) (ref.FC-102-1007) according to the manufacturer's instructions. Briefly, 2 μg of total RNA was incubated with magnetic oligo(dT) beads. Non poly-adenylated RNA was removed by several washes. Reverse transcription was performed on captured RNAs followed by the synthesis of the second strand of cDNA. Captured double stranded DNA was digested using DpnII. A ligation was performed with Illumina's GEX DpnII adapter 1 followed by a digestion using MmeI resulting in the release of tags. Those tags were ligated using Illumina's GEX Adapter 2, amplified by PCR (15 cycles) and purified on acrylamide gel. Libraries were validated using an Agilent DNA1000 BioAnalyzer chip, denatured using 0.1 N NaOH, diluted to 8 pM and sequenced on a Hiseq2000 using a Sequence by Synthesis technique.

#### Analysis and mapping of DGE tags

Image analyses and base-calling were conducted using the HiSeq Control Software (HCS 1.4.5.0) and RTA component (RTA 1.12.4.0). Extraction of 16 bp tags (reads were trimmed for adaptor sequence) and tag counting were performed using home-made Perl script.Sequences were first aligned (using the Illumina's sequencing analysis software, CASAVA 1.8) to transcripts of the *Apis mellifera* genome version 4.5 downloaded from NCBI. Only those 16 bp tags that were perfect matches were retained. Those tags that could not be aligned to transcripts were re-aligned to the complete *Apis mellifera* genome (version 4.5).

Mapping was also performed on sequences of honey bee virus genomes (Chronic bee paralysis virus RNA 1: GenBank EU122229, Chronic bee paralysis virus RNA 2: EU122230, Sacbrood virus: AF092924, Deformed wing virus: AJ489744, Black queen cell virus: AF183905, Acute bee paralysis virus: AF150629, Kashmir bee virus: AY275710, *Varroa destructor* virus 1: AY251269 and Israel acute paralysis virus: EF219380).

The package DESeq from Bioconductor was used to conduct the analysis [79]. Genes (i.e. tags that matched a transcript) and tags that were only aligned to the genome were analyzed separately. Tags that occur less than one time in a million, in two or more samples, were filtered from the analysis. DESeq estimates variance-mean dependence in count data from DGE-tag libraries and tests for differential expression based on a model using the negative binomial distribution. Genes were considered to be differentially expressed between two treatments at an adjusted *P*-value < 0.05. The *P*-value was adjusted for multiple testing with the Benjamini-Hochberg procedure, which controls for false discovery rate*.*

#### Analysis of gene expression profiles

Genes that overlapped between gene lists were identified by creating Venn diagrams using GeneVenn [80]. Exact hypergeometric probability test was performed to test the statistical significance of the overlap between two gene lists [81]. DAVID 6.7 [82,83] was used to determine the enriched functional groups, based on GO terms, within the complete list of expressed genes containing those genes with Flybase orthologs.

We also determined whether *Nosema* and *Varroa* modified the brain gene expression profile in a manner consistent with some previously characterized behavioral phenotypes or in a completely different way. To explore this idea, we compared the parasite effects to nurse/forager profiles that were obtained with microarray analysis [41], using Chi-square tests with Yates correction.

## Competing interests

The authors have declared no competing interests.

## Authors’ contributions

YLC, CMM and CA conceived of the study and its experimental design. CMM, DC and CA conducted the experimental protocol with the honey bees, ED and DB performed the chemical analysis, ED and CA performed the behavioral observations, CMM performed brain dissections and RNA isolation, HP and JPD carried out the sequencing and statistical analysis of sequencing data, CMM and CA analyzed and interpreted the data, and CMM, CA and YLC drafted manuscript. All authors read and approved the final manuscript.

## Supplementary Material

Additional file 1: Table S1 Relative proportion of each CHC compound in control and *Nosema* or *Varroa* parasitized bees. Changes in proportion induced by parasitism were determined with Mann–Whitney U tests (*P* < 0.05 are in bold). Click here for file

Additional file 2: Table S2S2 Percentage of correct assignments of *Nosema*-infected and control bees based on their cuticular hydrocarbons profiles. In the treatment column, 5 and 10 indicate the age of the bees. The last four columns show the number of bees classified in the different group treatment by the discriminant analysis. The experiment was repeated on bees from 3 different colonies. Table S3 Percentage of correct assignments of *Varroa*-infested and control bees based on their cuticular hydrocarbons profiles. The last six columns show the number of bees classified in the different group treatment by the discriminant analysis. The numbers 98, 120 and 231 indicate the colony origin of the bees. Click here for file

Additional file 3: Table S4 Summary of DGE sequencing results. The analysis was performed on two colonies. Click here for file

Additional file 4: Table S5 Lists of genes affected in the bee brain by *Nosema* or *Varroa* parasitism. Corresponding honey bee gene, *Drosophila* ortholog and genes also up- or downregulated in the brain of nurses and foragers are shown. Click here for file

Additional file 5: Table S6 Effects of *Nosema* and *Varroa* parasites on the prevalance of Deformed Wing Virus and *Varroa Destructor* Virus levels in the bee brain. Comparisons of viral titers (A) *Nosema* vs Control, (B) *Varroa* vs Control and (C) *Varroa* vs *Nosema* using Cox-Reid method for estimating dispersion and Generalized Linear Model (GLM) for statistical test. Click here for file
