# Supplementary material for: Ecto- and endoparasite induce similar chemical and brain neurogenomic responses in the honey bee (Apis mellifera)
Source: BMC Ecol. 2013 Jul 17;13:25. doi: 10.1186/1472-6785-13-25 (PMC3725162; doi:10.1186/1472-6785-13-25)
Supplement: Additional file 3: Table S4 — Summary of DGE sequencing results. The analysis was performed on two colonies. [file 1472-6785-13-25-S3.docx]

**Additional file 2.**

**Table S2. Percentage of correct assignments of *Nosema*-infected and control bees based on their cuticular hydrocarbons profiles.**

| Colony # | Treatment | % correct assignement | Control5 | *Nosema*5 | Control10 | *Nosema*10 |
| --- | --- | --- | --- | --- | --- | --- |
| Colony 98 | Control5 | 91.7 | 11 | 1 | 0 | 0 |
|  | *Nosema*5 | 100 | 0 | 12 | 0 | 0 |
|  | Control10 | 83.3 | 0 | 0 | 10 | 2 |
|  | *Nosema*10 | 100 | 0 | 0 | 0 | 11 |
|  |  |  |  |  |  |  |
| Colony 120 | Control5 | 91.7 | 11 | 1 | 0 | 0 |
|  | *Nosema*5 | 75 | 3 | 9 | 0 | 0 |
|  | Control10 | 83.3 | 0 | 0 | 10 | 2 |
|  | *Nosema*10 | 91.7 | 0 | 0 | 1 | 11 |
|  |  |  |  |  |  |  |
| Colony 231 | Control5 | 72.7 | 8 | 3 | 0 | 0 |
|  | *Nosema*5 | 91.7 | 1 | 11 | 0 | 0 |
|  | Control10 | 83.3 | 1 | 0 | 10 | 1 |
|  | *Nosema*10 | 83.3 | 0 | 0 | 2 | 10 |

**Table S3: Percentage of correct assignments of *Varroa*-infected and control bees based on their cuticular hydrocarbons profiles.**

| Treatment | % correct assignement | Control98 | *Varroa*98 | Control120 | *Varroa*120 | Control231 | *Varroa*231 |
| --- | --- | --- | --- | --- | --- | --- | --- |
| Control98 | 91.7 | 11 | 0 | 0 | 0 | 0 | 1 |
| *Varroa*98 | 75 | 0 | 9 | 1 | 0 | 1 | 1 |
| Control120 | 91.7 | 0 | 0 | 11 | 1 | 0 | 0 |
| *Varroa*120 | 100 | 0 | 0 | 0 | 12 | 0 | 0 |
| Control231 | 83.3 | 0 | 1 | 1 | 0 | 10 | 0 |
| *Varroa*231 | 75 | 3 | 0 | 0 | 0 | 0 | 9 |
