# Supplementary material for: Ecto- and endoparasite induce similar chemical and brain neurogenomic responses in the honey bee (Apis mellifera)
Source: BMC Ecol. 2013 Jul 17;13:25. doi: 10.1186/1472-6785-13-25 (PMC3725162; doi:10.1186/1472-6785-13-25)
Supplement: Additional file 4: Table S5 — Lists of genes affected in the bee brain by Nosema or Varroa parasitism. Corresponding honey bee gene, Drosophila ortholog and genes also up- or downregulated in the brain of nurses and foragers are shown. [file 1472-6785-13-25-S4.docx]

**Additional file 3. Table S4. Summary of DGE sequencing results.**

|  | Total tags | Total filtered tags | Total tags excluding adaptor sequence | Total aligned tags | Distinct aligned tags | Number of unique honey bee gene hits |
| --- | --- | --- | --- | --- | --- | --- |
| Control 1 | 102,262,143 | 90,806,353 | 89,764,576 | 814,072 | 81,521 | 9,341 |
| Control 2 | 115,478,265 | 100,102,121 | 99,004,989 | 844,021 | 81,524 | 9,342 |
| *Nosema* 1 | 105,762,567 | 92,617,425 | 91,427,020 | 783,372 | 77,019 | 9,295 |
| *Nosema* 2 | 108,958,773 | 95,024,700 | 93,286,400 | 865,433 | 81,190 | 9,355 |
| *Varroa* 1 | 107,912,845 | 93,139,798 | 92,028,921 | 701,757 | 76,121 | 9,299 |
| *Varroa* 2 | 107,765,170 | 93,710,619 | 92,405,878 | 729,935 | 76,656 | 9,318 |
