# Supplementary material for: Ecto- and endoparasite induce similar chemical and brain neurogenomic responses in the honey bee (Apis mellifera)
Source: BMC Ecol. 2013 Jul 17;13:25. doi: 10.1186/1472-6785-13-25 (PMC3725162; doi:10.1186/1472-6785-13-25)
Supplement: Additional file 5: Table S6 — Effects of Nosema and Varroa parasites on the prevalance of Deformed Wing Virus and Varroa Destructor Virus levels in the bee brain. Comparisons of viral titers (A) Nosema vs Control, (B) Varroa vs Control and (C) Varroa vs Nosema using Cox-Reid method for estimating dispersion and Generalized Linear Model (GLM) for statistical test. [file 1472-6785-13-25-S5.docx]

**Additional file 5.** **Table S6.** **Effects of *Nosema* and *Varroa* parasites on the prevalance of Deformed Wing Virus and *Varroa* *Destructor* Virus levels in the bee brain.**

A.

|  | Control vs *Nosema* | | | |
| --- | --- | --- | --- | --- |
| Virus | LogFC^1^ | logCPM^2^ | Likelihood Ratio | *P*-value |
| Deformed Wing Virus | 5.17 | 11.55 | 3.78 | 0.051 |
| Varroa destructor virus | 5.29 | 17.16 | 0.72 | 0.39 |

^1^log Fold change

^2^log Counts per million

B.

|  | Control vs *Varroa* | | | |
| --- | --- | --- | --- | --- |
| Virus | LogFC | logCPM | Likelihood Ratio | *P*-value |
| Deformed Wing Virus | 7.66 | 13.2 | 6.59 | 0.01 |
| Varroa destructor virus | 6.46 | 17.93 | 1.73 | 0.19 |

C.

|  | *Varroa* vs *Nosema* | | | |
| --- | --- | --- | --- | --- |
| Virus | LogFC | logCPM | Likelihood Ratio | *P*-value |
| Deformed Wing Virus | 2.45 | 13.57 | 8.96 | 0.003 |
| Varroa destructor virus | 1.12 | 18.19 | 2.27 | 0.13 |
